# Eco-Friendly Alternative Disposal through the Pyrolysis Process of Meat and Bone Meal

**DOI:** 10.3390/ma15196593

**Published:** 2022-09-22

**Authors:** Anca Maria Zaharioiu, Claudia Şandru, Eusebiu Ilarian Ionete, Florian Marin, Roxana Elena Ionete, Amalia Soare, Marius Constantinescu, Felicia Bucura, Violeta-Carolina Niculescu

**Affiliations:** National Research and Development Institute for Cryogenic and Isotopic Technologies—ICSI Ramnicu Valcea, Uzinei Street no. 4, 240050 Ramnicu Valcea, Romania

**Keywords:** alternative fuels, catalysts, meal and bone meal (MBM), mesoporous silica, pyrolysis, selective adsorbent materials

## Abstract

The capitalization of agri-food waste is essential for the sustainability of a circular economy. This work focuses on a solution to eliminate such waste, meat and bone meal (MBM), which is produced in large quantities by the food industry and is prohibited for use as animal feed under the European directives. Therefore, with the focus of converting waste to energy, the catalytic pyrolysis of MBM in the presence of mesoporous silica nanocatalysts (SBA-3 and SBA-16 materials and metallic derivates) was investigated in a home-made reactor for the production of renewable energy. The mesoporous silica materials were synthesized using relatively simple methods and then characterized in order to determine their morpho-structural characteristics. The MBM pyrolysis behavior under different experimental conditions was examined in detail, both in the presence and absence of the new catalysts. The resulting MBM-based pyrolysis products, MBM_PYOIL_s and MBM_PYGAS_s, were also assessed as potential alternative fuels, highlighting comparable energy values to conventional fuels. The outcomes of this investigation offer a potential pathway to the clean production of gas and oil, thus promoting the high-grade utilization of MBM waste.

## 1. Introduction

It is undeniable that the world is currently at a critical juncture in terms of climate change. The process is irreversible and has serious consequences. High levels of seas and oceans will result from global temperatures rising by more than 2 °C, and this is only one of the repercussions of climate change. The origin of these events is mostly due to man’s unreasonable exploitation of resources. The capacity of using waste as a raw material to create energy might lead to the development of new alternative fuels and selected adsorbent materials, implying responsibility, better resource management, sustainability, and durability.

The possibility of using, through a novel concept of disposal by recovery, various waste raw materials for green energy purposes was investigated in this work, including aspects of both research and development. With the stated goal of creating alternative fuels through complex pyrolysis processes, the current study attempts to overlap numerous relevant concerns, namely (i) resource sustainability, (ii) environmental protection, and (iii) disposal—as complementing subjects [1,2,3].

Meat and bone meal (MBM) are a food industry waste generated in substantial amounts (14.2 Mt/y in the E.U. [4]) and prohibited to be used as animal feed according to Regulation (EC) No. 999/2001. The rationale for this is that MBM from cattle, sheep, and goats is regarded a high-risk material for bovine spongiform encephalopathy (BSE) [5,6,7]. BSE (bovine spongiform encephalopathy) is a progressive neurological disorder of cattle that results from infection by an unusual transmissible agent called a prion. The nature of the transmissible agent is not well understood [5]. Currently, the most accepted theory is that the agent is a modified form of a normal protein known as prion protein. For reasons that are not yet understood, the normal prion protein changes into a pathogenic (harmful) form that then damages the central nervous system of cattle. As a result of the recent bovine spongiform encephalopathy (BSE) crisis in the European beef industry, the use of animal by-product is now severely controlled [6]. Meat and bone meal (MBM) production can no longer be used to feed cattle and must be safely disposed of or transformed. The sustainability of MBM resources for pyrolysis processes cannot suffer from the point of view of continuity, since this waste is a continuous by-product of the food industry, being processed from the remains after boning—ligaments, bones, skin, bone marrow and traces of meat [7].

Currently, countries are adopting landfilling as a method of removing MBM. Another solution for eliminating MBM is combustion or incineration, although the ultimate one is ash generating, emitted from incineration plants and can also lead to the formation of furans and dioxins, if such plants do not meet the required parameters of, respectively, T = 850 °C for t = 2.5 s [8]. MBM, a by-product of the meat-processing industry, is a mixture of 48–52% protein, 33–35% ash, 8–12% fat, and 4–7% moisture [9]. MBM is processed from scrap after deboning—ligaments, bones, skin, bone marrow and traces of flesh [10]. In the past, MBM has been used on a global scale mainly for animal feed, to improve the amino acid profile of the feed. Recent years have been characterized by the growing interest in the sustainability of resources, this being transposed in the research related to the co-combustion of fossil fuels (e.g., lignite, coke, coal, anthracite, coke dust, peat, fuel oil, and natural gas) mixed with various types of biomass waste [11,12,13,14]. MBM has also been the subject of worldwide research since such waste from the meat-processing industry was proved to be hazardous to both the physical and mental health of animals. Although MBM flour is rich in calcium and phosphorus, nutritionists believe that this can upset the body’s calcium and phosphorus balance [15,16]. Therefore, MBM has been eliminated from animal feed in several states in the EU, USA, Japan, and Brazil. Under the conditions described above, MBM, which is now, at least in theory, a waste for today’s modern society, requires a feasible solution for capitalization. Due to its organic nature, with contents of the combustible elements C (36 wt.%), H (5.8 wt.%) and O (15.3 wt.%), respectively, with heating values between 17 and 20 MJ/kg, MBM looks suitable to generate thermal and electrical energy through specific processes and technologies, such as combustion, co-combustion, gasification, and incineration, and thus is assimilated to the energy produced by biomass [17]. In the Nordic countries, but also in Netherlands and Germany, MBM is frequently used to produce energy by combustion in incinerators, alone or together with other wastes, and fuel matrices, especially for energy-intensive industries such as building materials, cement industry, and asphalt production [18]. Pyrolysis and gasification of MBM were also evaluated with promising yields, obtaining both liquid and gaseous fractions, with high energy value, comparable to those of conventional fuels [19,20,21,22]. Additionally, the energy efficiency of the conventional biomass residue mixture from the olive oil preparation with MBM was tested in a fluidized bed system, together with an emissions investigation according to the operating conditions and an environmental impact assessment. The results showed low CO emissions and negligible SO_2_ emissions, while NO_x_ emissions did not exceed the limits of EU legislation [23,24]. Another advantage of such mixtures, in addition to the disposal of MBM due to the high energy value obtained, is that the left residue at the end of such processes, the ash, could be used successfully to improve eroded and degraded soils or in the building materials industry (e.g., to bricks manufacturing) due to the high level of metals such as Ni, Ca, Zn, and Mg [25,26]. A component of various solid-energy-generating matrices, MBM, through combinations with coal or biomass, is a friendly solution of elimination by energy recovery for environment and quality of life [27,28], and this work confirms these aspects through experimental data. The choice of MBM for this study is related to the addition of this type of waste with biomass, and in the case of combustion or co-combustion of pyrolysis products, the carbon footprint is neutral [29]. Other food waste (rice, vegetables, and fish) was blended in definite ratios (70:30, 60:40, and 50:50 w/w) with polyethylene terephthalate (PET) to develop a process for producing bio-oil, char, and value-added chemicals using co-pyrolysis under controlled conditions [30].

Mesoporous molecular sieves such as SBA-3 can be synthesized at room temperature under acidic conditions, similar to SBA-15 synthesis, except the template may be a low-molecular-weight quaternary alkylammonium. SBA-3 (with a pore diameter > 3 nm) may also contain micropores [31]. The development of this material is interesting for applications in catalysis at high temperatures. SBA-16 is a porous silica with large (5–15 nm) cage-like mesopores arranged in three-dimensional centered cubic symmetry [28]. Like SBA-15, it is synthesized under acidic conditions using a non-ionic surfactant—Pluronic—providing complementary porosity [32]. The mesophase can be created using mixtures of Pluronic P123 and Pluronic F127. SBA-16 generally has thicker pore walls than SBA-15. As suggested by electron crystallography studies [32], each mesopore is connected to eight neighboring mesopores, making this feature the limiting factor for applications involving intra-particle mass transfer.

The purpose of this study was the eco-friendly application of the new nanomaterials as catalysts for the pyrolysis process of meat and bone meal (MBM), in order to dispose this waste and obtain valuable products, such as energy and new adsorbents, in terms of a circular economy. With the focus of converting waste to energy, the catalytic pyrolysis of MBM integrated by SBA-3 and SBA-16 transitional-metal derivates was investigated in a home-made reactor for the production of renewable energy. The MBM pyrolysis behavior under different experimental conditions was examined in detail. The outcomes of this investigation offer a potential pathway to the clean production of gas and oil, thus promoting the high-grade utilization of MBM waste.

## 2. Materials and Methods

### 2.1. MBM Preparation

The MBM used in this study is a mixture of meat and bones of several types, namely: poultry meat and bones, meat and beef bones, and pork meat and bones, in equal ratio in the feedstock blend. A major impediment to the disposal of this waste by energy recovery is the moisture content, which can vary from 12 wt.% to 25 wt.%. Therefore, the use of an energy-efficient MBM dehumidification solution [33,34] can lead to high efficiencies of future combustion or pyrolysis processes.

Meat and bones were dehydrated/dried at T = 105 °C, t = 8 h, until constant mass, in an oven without ventilation (Nahita 601, London, UK). A moisture content (W_final_) of almost 5 wt.% was obtained, compared to the W_initial_ ~23 wt.%. After the dehydration/drying process, the obtained mixture was ground (Cutting Mill Pulverisette 15, FRITSCH GmbH–Milling and Sizing, Weimar, Germany) at dimensions of <200 μm. In total, more than 2 kg of meat and bones was processed, in a ratio of 70%:30%.

### 2.2. Catalysts Preparation

The SBA-16 support was prepared following a previously developed method [35]. Briefly, 1.5 g Pluronic P127 (Sigma Aldrich, Darmstadt, Germany) was dissolved (under magnetic stirring at ambient temperature) in 72 mL ultrapure water and 7 mL 37% HCl (Sigma Aldrich, Darmstadt, Germany). After 30 min, 6 mL butanol as co-surfactant (Sigma Aldrich, Darmstadt, Germany) and 7.5 mL tetraethyl orthosilicate (TEOS-Sigma Aldrich, Darmstadt, Germany) were added under continuous stirring, with increasing temperature up to 45 °C for 24 h. The mixture was then transferred to a Teflon autoclave for hydrothermal treatment at 100 °C for 24 h, the solid being collected by filtration, dried and calcined at 550 °C for 6 h, in order to remove the residual surfactant. The support, SBA-16 (0.3 g), was impregnated with aqueous solution of nickel acetate (1.5 mL solution of concentration 1.34 % Ni) (Sigma Aldrich, Darmstadt, Germany). After impregnation, the precursor was dried for 24 h in air and then calcined at 550 °C in air for 6 h. To obtain the iron catalyst, 0.375 g SBA-16 was treated with 6.25 mL 1 M NaCl (Sigma Aldrich, Darmstadt, Germany); then, 25 mL 20 mM FeCl_3_ solution (Sigma Aldrich, Darmstadt, Germany) was added, with the mixture being made up to 500 mL with ultrapure water, under stirring. The pH was measured to be around 3 and the mixture was left under stirring for 24 h at room temperature. After this interval, the pH was checked again and allowed to settle in a separating funnel for 24 h. The mixture was centrifuged, the supernatant was removed and the brick-colored precipitate obtained was dried at 100 °C for 5 h.

The SBA-3 support was prepared following a previously developed method [36]. SBA-3 mesoporous silica was prepared using cetyltrimethylammonium bromide (Sigma Aldrich, Darmstadt, Germany) and tetraethyl orthosilicate (Sigma Aldrich, Darmstadt, Germany) as template and source of Si, respectively. An aqueous solution of HCl (37%) was added to control the pH of the system reaction. Thus, 1 g cetyltrimethylammonium bromide and 40 mL HCl (37%) were dissolved in 100 mL ultrapure water. TEOS (10 mL) was added dropwise to the acidic solution under vigorous stirring at 30 °C. After 2 h, the white precipitate (SBA-3 precursor) was aged at room temperature for 12 h. The sample was then filtered and dried for 12 h at 100 °C. SBA-3 was then immersed at reflux in ethanol for 6 h to extract the surfactant; after that, the precipitate was filtered and washed with ultrapure water. After drying, SBA-3 mesoporous silica was calcined at 550 °C in air for 6 h. The SBA-3 derived catalysts were prepared according to the method for SBA-16-derived catalysts.

### 2.3. Characterization Methods

The inquiry methodologies employed in this study yielded qualitative and quantitative investigation for the used waste, namely MBM, the used catalysts, as well as for the products resulting from pyrolysis procedures (oil—MBM_PYOIL_, gas—MBM_PYGAS_, solid residue, char—MBM_PYCHAR_ and the developed selective adsorbents).

The specific surface areas and pore distribution of all the obtained materials were determined using BET (Brunauer–Emmett–Teller) and BJH (Barrett–Joyner–Halenda) procedures using Quantachrome Autosorb-IQ porosity equipment (Quantachrome Instruments, Boynton Beach, FL, USA) [37]. Prior to analysis, the samples were degassed at 150 °C.

The morphology of both catalysts and resulting adsorbents was investigated using scanning electron microscopy, using a FESEM VP Scanning Electron Microscope (Carl Zeiss, Oberkochen, Germany), at a resolution of 0.8 nm and 2.5 nm VP mode, at 30 kV.

Functional groups of catalysts and pyrolysis oil were highlighted using FTIR analysis (Cary 630 ATR-FTIR spectrophotometer—Agilent Technologies, Inc., Santa Clara, CA, USA). Prior to analysis, the solid samples were ground in an agate mortar and dried at 80 °C under vacuum, to avoid the appearance of physically adsorbed water. The pyrolysis oil was pipetted directly on the ATR diamond. The spectra acquisition was achieved by collecting spectra between 4000 and 400 cm^−1^ (32 scans for background and samples at 8 cm^−1^ resolution and 0.002 threshold). The spectra were interpreted with the help of MicroLab Expert v.1.0.0 Software available from Cary 630 ATR-FTIR spectrophotometer (Agilent Technologies, Inc., Santa Clara, CA, USA).

The elemental analysis was performed by using the combustion and the pyrolysis methods coupled with the gas chromatography method (Flash EA2000, Thermo Scientific, Waltham, MA, USA) [38]. The metal content was determined using an atomic absorption spectrophotometer (NovAA 300, Analytik Jena AG, Jena, Germany).

The resulting pyrolysis gases analysis was carried out using gas chromatography, using the instrument GC Varian CP 3800 (Palo Alto, CA, USA). The method was developed using the natural gas investigation standard methods [39,40], in order to identify and quantify permanent gases (CO, CO_2_, H_2,_ O_2_, and N_2_) and hydrocarbon gases (isomers C1-C9). Additionally, the physical characteristics that were investigated were, respectively, the upper and lower calorific value, the density, and the Wobbe number using previously developed methods [41].

Both initial MBM and resulting MBM_PYCHAR_ were subjected to calorimetric investigations, using IKA C5000 calorimeter (IKA, Staufen, Germany) in order to determine their energy values. A quantity of about 1 g was used in the oxygen atmosphere bomb [38]. The solid samples were previously dried, ground under 0.2 mm, sieved and loaded into the quartz platform. The resulting MBM_PYOIL_, around 0.2 g/sample, was also investigated to determine the energy potential [42,43].

Since human exposure to various chemicals that may affect the endocrine and/or carcinogenic systems is a major concern, the risk to human health posed by waste such as MBM has to be considered. Therefore, the equivalent total toxicity concentration (TEC), defined as the concentration of individual congener dioxins/furans, mixed carcinogens multiplied by their relative power factors or toxic equivalence factors, was first addressed. For that, the most common and toxic dioxin 2,3,7,8 tetrachlorodibenzo-dioxin (TCDD), with a toxic equivalence factor (TEF) of 1 [41], was used as a reference, and results on polychlorinated dibenzo-p-dioxins (PCDDs)/polychlorinated dibenzo-p-furans (PCDFs) concentrations in MBM, but also the equivalent TEC toxicity concentration, were assessed [42,43,44].

The investigated MBM had an equivalent toxic concentration (TEC) for PCDDs of 0.790 ng/kg, and of 0.213 ng/kg for PCDFs, respectively. For a total TCDD of 1.003 ng/kg, the value is below that set for the cleaning level, 13.000 ng/kg. According to previous studies [43,44], there is no risk of handling this waste or the produced reaction products (MBM_PYGAS_, MBM_PYOIL_, and MBM_PYCHAR_) with this cleaning level for 2,3,7,8-TCDD in MBM.

### 2.4. Pyrolysis Process

**Figure 1:**
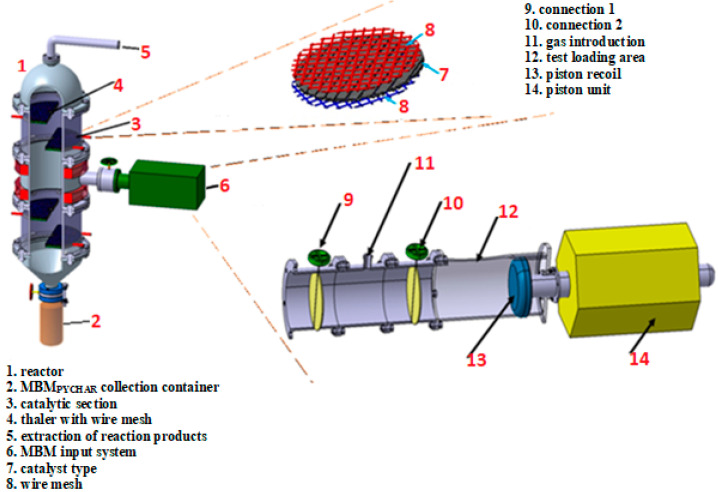
Flowsheet of the MBM’s experimental pyrolysis plant.

Approximatively 50 g of dry MBM was used during the pyrolysis process. The inert atmosphere of the pyrolysis processes was achieved under high-purity nitrogen, 99.999 %vol (Messer Magnicom Gas, Ramnicu Valcea, Romania). The flow reactor benefited from an automatic temperature control system, PID controller, and a type K thermocouple arranged in a steel jacket inside of it. The temperature from which the pyrolysis reactor started was almost 23 ± 0.5 °C; the ambient temperature and the feed rate used in the pyrolysis process was 5 °C/min until the flow reactor reached the final temperature of 450 °C. The total time of an experiment was about 2 and a half hours: 1 h and a half until the reactor reached the final temperature of 450 °C and 1 h for the pyrolysis process itself. Water, at 10 °C, was used as a cooling agent for the process, without being contaminated following the experimental pyrolysis tests. The inert gas flow used in the pyrolysis processes was constant, ~100 mL/min, with insignificant variations of ±5%. In order to regulate the inert gas flow, a two-stage pressure regulator (Linde Gas, Pullach im Isartal, Germany) with fine pressure regulation was used for the gas cylinder (V = 30 L). The MBM’s pyrolysis testing stand based on a patented configuration [38,44] consisted of: (i) single fluidized bed reactor; (ii) condenser; (iii) non-condensable gas collection system; and (iv) condensate gas collection system (Figure 1).

## 3. Results and Discussions

### 3.1. MBM Toxicity

Since human exposure to various chemicals that may affect the endocrine and/or carcinogenic systems is a major concern, the risk to human health posed by waste such as MBM has to be considered. Therefore, the equivalent total toxicity concentration (TEC), defined as the concentration of individual congener dioxins/furans, mixed carcinogens multiplied by their relative power factors or toxic equivalence factors, was first addressed. For that, the most common and toxic dioxin 2,3,7,8 tetrachlorodibenzo-dioxin (TCDD), with a toxic equivalence factor (TEF) of 1 [45], was used as a reference, and results on *Polychlorinated dibenzo-p-dioxins* (PCDDs)/*Polychlorinated dibenzo-p-furans* (PCDFs) concentrations in MBM, but also the equivalent TEC toxicity concentration, were assessed [46,47,48].

The investigated MBM had an equivalent toxic concentration (TEC) for PCDDs of 0.790 ng/kg, and of 0.213 ng/kg for PCDFs, respectively. For a total TCDD of 1.003 ng/kg, the value was below that set for the cleaning level, 13.000 ng/kg. According to previous studies [47,48], there is no risk of handling this waste or the produced reaction products (MBM_PYGAS_, MBM_PYOIL_, and MBM_PYCHAR_) with this cleaning level for 2,3,7,8-TCDD in MBM.

### 3.2. SBA-16 Support and Catalysts’ Characterization

**Figure 2:**
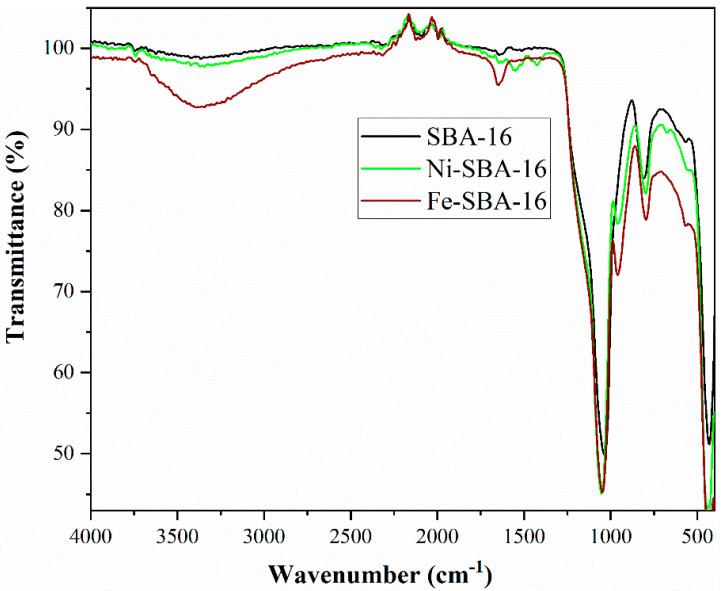
FTIR spectra for SBA-16 support and catalysts.

The typical FT-IR spectra for SBA-16 and catalysts (Figure 2) show the absorption bands characteristic of nanostructured silica, with a broadband attributed to asymmetric Si-O-Si vibration at 1050 cm^−1^. In addition, a weak band at 800 cm^−1^ assigned to the symmetrical tensile vibration for Si-O bonds can be observed.

It can be observed that at 3340 cm^−1^, the SBA-16 support has bands specific to the presence of OH groups on the surface of the silanol groups or to the water molecules adsorbed on the surface. After functionalization with metals, the bands’ intensity decreases, which proves the immobilization of the metal on the surface. The absorption band at 1658 cm^−1^ of the SBA-16 sample can be attributed to the deformation vibrations of the adsorbed water molecules (δ_H-O-H_). The approximately 950 cm^−1^ bands in the spectra of the two catalysts are assigned Si-OMe [49].

**Figure 3:**
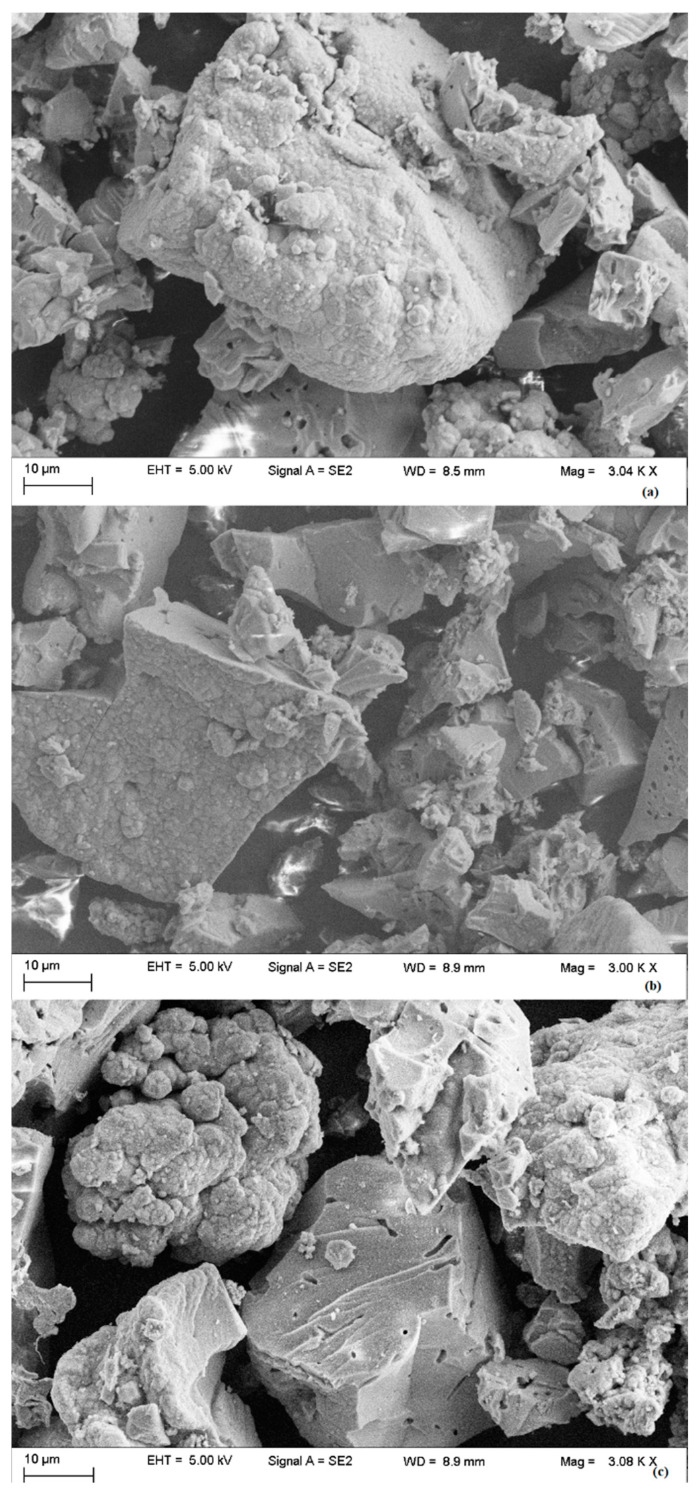
SEM images for SBA-16 mesoporous silica (**a**), Ni (**b**) and Fe (**c**) catalysts.

Figure 3 shows the SEM images of SBA-16 mesoporous silica and Ni and Fe catalysts.

The size and shape of the particles indicate a good morphology of the crystals, without other phases, typical for these materials. As can be seen in Figure 3, almost-spherical crystals were separated, and for the catalyst samples, the deposition of the metal on the silica particles can be observed (Ni—Figure 3b; Fe—Figure 3c).

**Figure 4:**
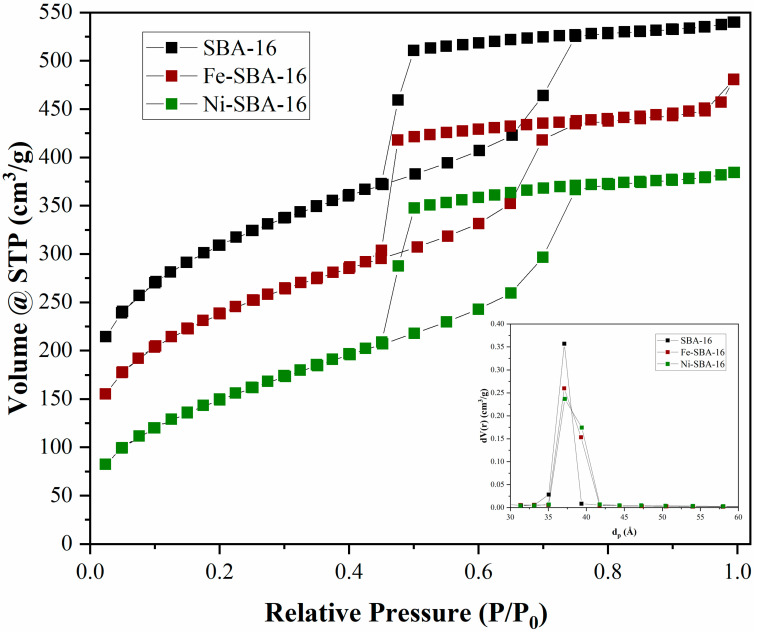
Adsorption–desorption isotherms and pore diameters for SBA-16 group.

The BET and pore size distribution are in agreement with the literature data for such materials (Figure 4).

For SBA-16 and the two catalysts, the isotherms are type IV, according to the IUPAC definition, associated with the presence of mesopores [50]. SBA-16 contains asymmetric and triangular adsorption and desorption branches and these characteristics can be attributed to systems with pore network connectivity and a blocking effect with a H_2_ hysteresis loop. As expected, the introduction of metals leads to a decrease in surface area. The specific surfaces were 911 m^2^/g for SBA-16, 436 m^2^/g for Ni-SBA-16, and 656 m^2^/g for Fe-SBA-16. The average pore diameter was around 3.7 nm for all three samples.

Elemental analysis and EDX revealed a content of 53 wt.% by weight Si for SBA-16, 42 wt.% for Ni-SBA-16, and 40 wt.% for Fe-SBA-16 (Table 1). The metal was also identified and quantified by EDX and atomic absorption spectrometry (SAA) (Table 1).

### 3.3. SBA-3 Support and Catalysts Characterization

**Figure 5:**
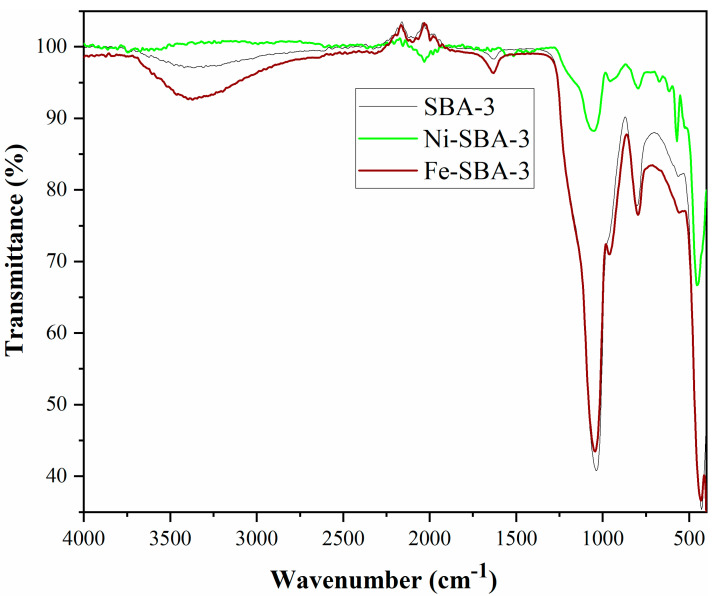
FTIR spectra for SBA-3 support and catalysts.

The typical FT-IR spectra for SBA-3 and Ni and Fe catalysts (Figure 5) show the absorption bands characteristic to nanostructured silica, with a broadband attributed to asymmetric Si-O-Si tensile vibration at 1050 cm^−1^. In addition, a weak band at 800 cm^−1^ assigned to the symmetrical tensile vibration for Si-O bonds can be observed.

It can be observed that at 3435 cm^−1^, the SBA-3 support has bands specific to the presence of OH groups on the surface of silanol groups or to water molecules adsorbed on the surface. After functionalization with metals, the bands’ intensity decreases, which proves the immobilization of the metal on the surface. The absorption band at 1650 cm^−1^ of the SBA-3 sample can be attributed to the deformation vibrations of the adsorbed water molecules (δ_H-O-H_). The approximately 960 cm^−1^ bands in the spectra of the two catalysts are assigned to Si-OMe [49].

**Figure 6:**
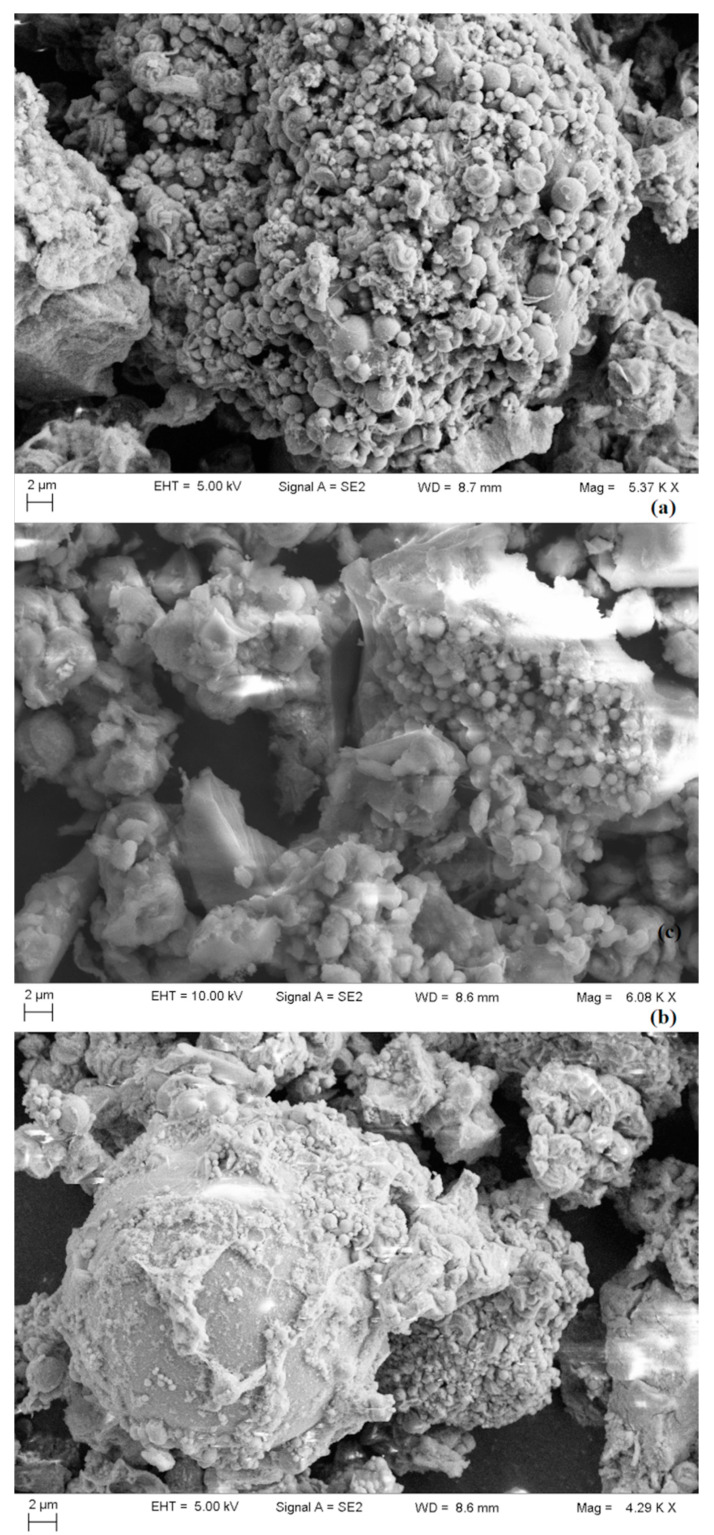
SEM images for SBA-3 mesoporous silica (**a**), Ni (**b**) and Fe (**c**) catalysts.

Figure 6 shows the SEM images for SBA-3 mesoporous silica and catalysts.

**Figure 7:**
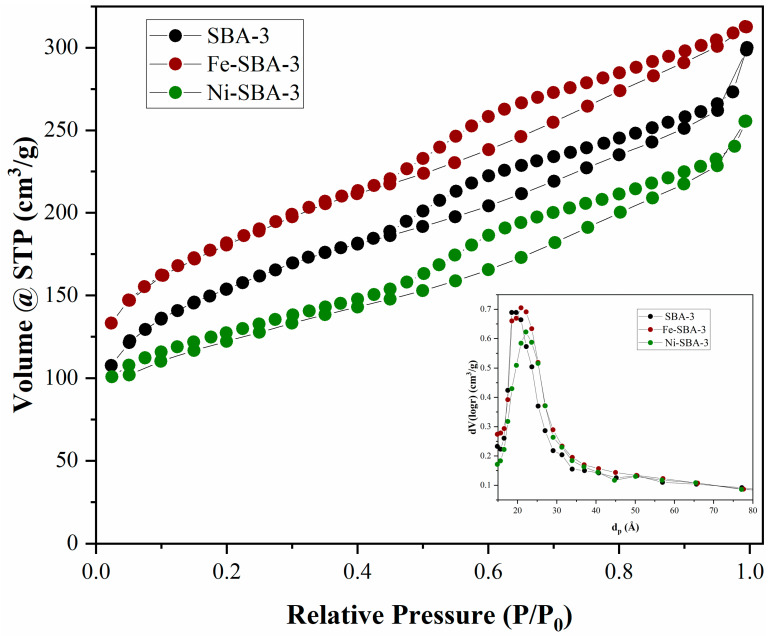
Adsorption–desorption isotherms and pore diameters for SBA-3 group.

For SBA-3 and the two catalysts, the isotherms are type I according to the IUPAC definition (Figure 7). The collapse of the hysteresis loop is indicated by the pronounced narrowing of the pore radius, which indicates the presence of mesopores. The specific surfaces were 549 m^2^/g for SBA-3, 268 m^2^/g for Ni-SBA-3, and 507 m^2^/g for Fe-SBA-3. The mean pore diameter was around 2.2 nm for all three samples (Figure 7).

Elemental analysis and EDX revealed a content of approximately 49 wt.% by weight Si for SBA-3, 40 wt.% for Ni-SBA-3, and 46 wt.% for Fe-SBA-3 (Table 2). The metal was also identified and quantified by EDX and atomic absorption spectrometry (SAA) (Table 2).

### 3.4. MBM_PYOIL_ Characteristics

MBM_PYOIL_ was studied to assess its energy potential as an alternative fuel, not only in comparison to other types of oils derived from the pyrolysis of various wastes, but also in comparison to other traditional liquid fuels such as fuel oil, diesel, and gasoline. Liquid samples obtained by pyrolysis processes, catalyzed (PPs) and uncatalyzed (UPs), have a high water content. The ratio of fuel oil vs. water is 3:1 in favor of the oil. Therefore, a separating funnel was used to remove water from the alternative fuel liquid, the MBM_PYOIL_ type oil. The appearance of this developed oil (MBM_PYOIL_) is specific, and it has a smell of hydrocarbons, a red-brown color, and is very flammable.

**Figure 8:**
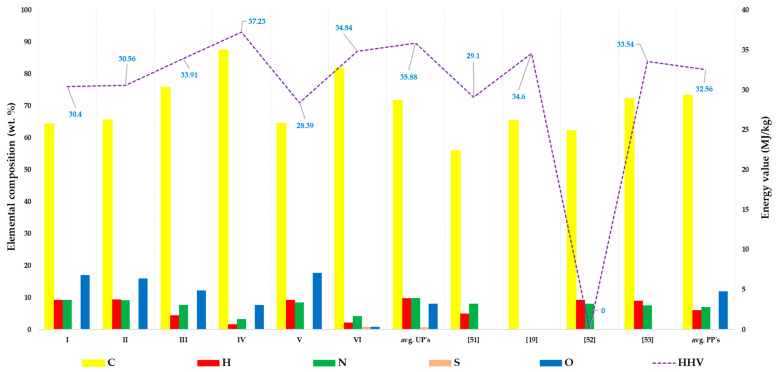
Comparative evaluation of physico-chemical characteristics of MBM_PYOIL_ obtained by pyrolysis in different experimental conditions compared with literature values [19,51,52,53].

The comparative analysis shows MBM_PYOIL_ was developed with a low water content, less than 1 wt.%, in relation to the range between 3.5 and 22 wt.% for MBM pyrolysis oils from the specialized literature [51,52,53]. The content of energy elements, carbon (C) and hydrogen (H), was slightly higher (Figure 8) than those mentioned in the literature, at 71.79 wt.% for C versus [56 wt.% to 72.2 wt.%] [51,52,53], and 9.82 wt.% for H compared to [5.0 wt.% to 9.2 wt.%] [51,52,53]. If future SO_x_ emissions are not a problem due to the low elemental sulphur (S) content, <1 wt.%, in theory, MBM_PYOIL_ can cause NOx emissions problems due to the high nitrogen (N) content, 10 wt.% (Figure 8), which is slightly higher than what the literature suggests, with data ranging from [7.5 wt.% to 8.1 wt.%] [52,53]. In most cases, the level of S content, and hence the prospective SO_x_ emissions, is less than 50 ppm [53]. The S content of oils produced with SBA catalysts ranged from [0.005 wt.% to 0.79 wt.%], similar to those found in non-catalytic processes, of 0.70 wt.%. In order to be readable, the samples were coded as follows: catalyzed pyrolytic oils (PPs) I—MBM_PYOIL(SBA-3)_; II—MBM_PYOIL(SBA-16_); III—MBM_PYOIL(Fe-SBA-3);_ IV—MBM_PYOIL(Fe-SBA-16)_; V—MBM_PYOIL(Ni-SBA-16)_; and VI—MBM_PYOIL(Ni-SBA-3)_.

The level of nitrogen that might generate NO_x_ emissions in the circumstance of the future combustion of developed oils is subject to the effect of catalysts in most cases; it is reduced in comparison to the level of N in non-catalytic processes, respectively, from 3.22 to 9.28 wt.% vs. 9.78 wt.%. The following catalysts were found to have the strongest effects in reducing nitrogen concentration: Fe-SBA-16 > Ni-SBA-3 > Fe-SBA-3 > Ni-SBA-16 > SBA-16 > SBA-3.

Catalytic pyrolysis with zeolites upgrades biomass pyrolysis products to hydrocarbons; however, they suffer from low yields due to high light-gas production and excessive catalyst coking. The explored silica catalysts have low acidity compared to zeolites; thus, they will partially deoxygenate pyrolysis products and can potentially form less coke. Furthermore, catalysts with low acidity can generate partially deoxygenated species such as furans, phenols, and cresols.

Regarding the average energy value of MBM_PYOIL_s produced in this work, it falls inside the domain within the scope described in the literature, of 34.22 MJ/kg versus the range [29.10 MJ/kg to 34.60 MJ/kg] [19,52,53]. Despite the fact that in absolute mean values, the lowest HHV (high heating value) belongs to MBM_PYOIL_s developed in catalyst-based pyrolytic processes, the highest energy value belongs to IV (37.23 MJ/kg) and the lowest to V (28.39 MJ/kg). The fact that MBMPYOIL* has the highest H_2_ concentration of 9.82 vol.% explains why its energy value is higher than the energy averages established by oils based on SBAs which are, respectively, 35.88 MJ/kg vs. 32.56 MJ/kg.

After a comparative analysis, it was observed that the content of heavy metals (Table 3) was lower than that of conventional fossil fuels [54,55,56]. As a result of the pyrolysis process, the initial metal levels in the MBM most likely migrated to the solid residue (Table 5).

**Figure 9:**
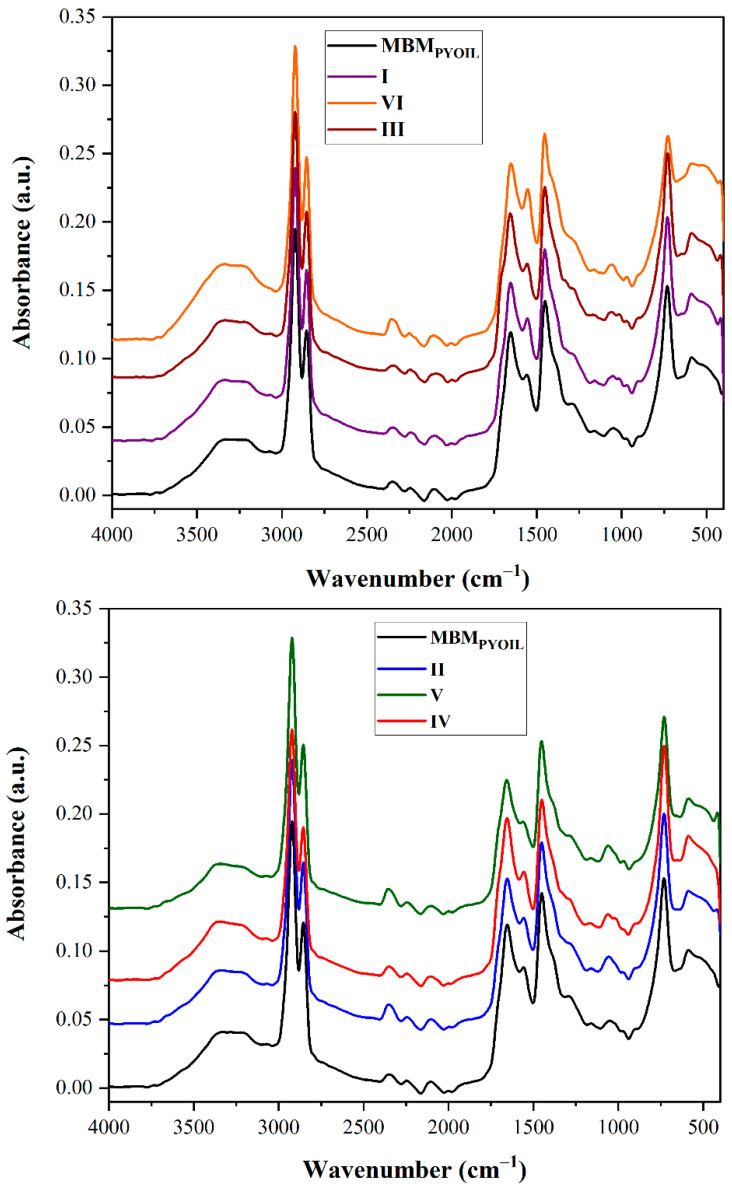
FTIR spectra of pyrolysis oil obtained in the absence and presence of catalysts ((**upper**) graph-SBA-3 group; (**bottom**) graph-SBA-16 group).

To determine the distribution of the functional organic groups in the pyrolysis oil samples, an FTIR characterization was performed (Figure 9).

The spectra were grouped so that the distribution took into account the mesoporous silica support. The upper part of Figure 9 presents the spectra of MBM_PYOIL_ which resulted from using SBA-3 (I), Fe-SBA-3 (III) and Ni-SBA-3 (VI). The bottom part of Figure 9 presents the spectra of MBM_PYOIL_ which resulted from using SBA-16 (II), Fe-SBA-16 (IV) and Ni-SBA-16 (V). For comparison reasons, in both graphs, the MBM_PYOIL_ resulting from the non-catalytic process was introduced. Interpretation of the FTIR spectra of pyrolysis oils occurring in the presence of Fe catalysts concludes that there are various peaks with high, medium, wide and low intensities, which indicate different types of connections, such as C-H, C-O, C-O, O-H, N-H and C-N in the fractions of oil (Figure 9) [58]. C-O stretching vibrations between 2000 cm^−1^ and 2366 cm^−1^ are due to ketones and aldehydes. Similarly, bands between 1155 cm^−1^ and 2159 cm^−1^ represent C-C stretching vibrations due to the presence of alkenes and aromatic compounds. The 3294 cm^−1^ band indicates the presence of groups of aromatic rings. The stretching vibrations of C-H from 2322 cm^−1^ and 2637 cm^−1^, as well as the deformation vibrations between 1380 cm^−1^ and 1457 cm^−1^, indicate the presence of alkanes. The appearance of both O-H groups and C-O stretching vibrations indicates the presence of carboxylic acids and their derivatives in pyrolysis oil [58]. It was concluded that the presence of different functional groups indicates the existence of hydrocarbons in the oil, and also the wide-peak O-H indicates the presence of water content in the oil. The wide-peak O-H is due to the interaction (hydrogen binding) of water molecules. Thus, the resulting oils contain polyamides, as well as alkanes, alkenes, ethers, alcohols and aromatic compounds. The FTIR spectra of pyrolysis oils that resulted in the presence of Ni catalysts show similarities with the spectra of pyrolysis oils developed on the basis of Fe catalysts, respectively; the same main peaks, with slightly modified intensities; as well as possible minor displacements.

### 3.5. MBM_PYGAS_ Characteristics

**Figure 10:**
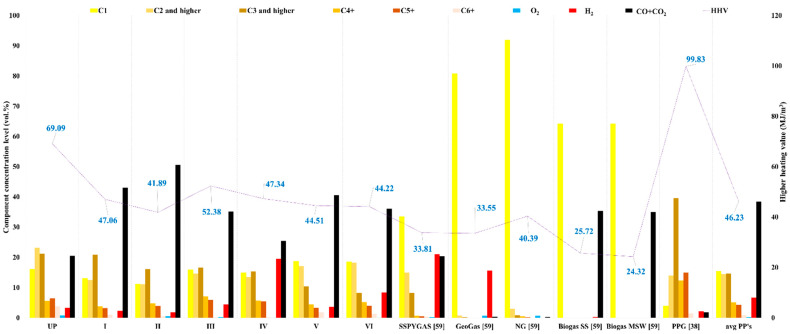
Comparative evaluation of physico-chemical characteristics for MBM_PYGAS_ obtained by pyrolysis in different experimental conditions compared with values from literature [38,59].

The gas formed as the initial reaction product from the complex pyrolysis processes was collected and investigated in terms of composition (qualitative and quantitative), permanent gases, and hydrocarbons, respectively, in terms of energy properties and environmental impact. As a physical feature, in a proportion of 99%, the resulting pyrolytic gases reveal specific properties, respectively, of high viscosity, whitish-gray color with the period of occurrence in the thermal range (from 270 °C to 450 °C), unpleasant smell and H_2_S content. All pyrolytic processes, as well as GC type investigations, were performed in triplicate, RSD < 1%. The presence of condensation/oily liquid on the inside walls of the Tedlar bag used to sample the MBM_PYGAS_ led to the assumption that several condensation columns would have been required for these operations, with the process benefiting from three columns, one metallic and two serpentine glasses. The experiments were carried out in a N_2_ atmosphere, and in the pyrolytic gas mixture, the content varied between 63 and 78 %vol. Thus, in the calculation of the final compositional concentrations of the pyrolytic gases developed for this study, respectively, of the corresponding energy calculation, the N_2_ content was subtracted and the compositions were normalized to 100%. GC-TCD/SM5A/Porapak Q investigations of MBM-based pyrolytic gases showed a high CO_2_ content, from 16.76 to 51.38 vol.%, while through GC-FID/Al_2_O_3_/KCl, PY_GASES_ had a varied hydrocarbon content (Figure 10). The samples were coded as follows: catalyzed pyrolytic gases—PP’s): I—MBM_PYGAS(SBA-3)_; II—MBM_PYGAS(SBA-16)_; III—MBM_PYGAS(Fe-SBA-3)_; IV—MBM_PYGAS(Fe-SBA-16)_; V—MBM_PYGAS(Ni-SBA-16)_; VI—MBM_PYGAS(Ni-SBA-3)_; SS_PYGAS_—sewage sludge pyrolysis gas; GeoGas—geothermal gas; NG—natural gas; BiogasSS—sewage sludge biogas; BiogasMSW—municipal solid waste biogas; and PPG—plastic pyrolysis gas (HDPE). Investigations regarding the composition of the component gases were carried out by ICSI Râmnicu Vâlcea [38,59].

The depletion of hydrocarbon levels and free radical movements (e.g., -CH3 and -H) is noticeable (both for MBM-based gases and under the action of catalysts) as a result of the reactions developed in the pyrolysis process (e.g., cracking, hydrogenation, dehydrogenation, cyclization, etc.), ultimately influencing qualitatively/energetically the product MBM_PYGAS_ [38]. An example of the influence of both reactions inside the pyrolysis process is MBM_PYGAS_, with high levels of concentrations for C2 and higher of 18.91 vol.%, and C3 and higher of 21.12 vol.%, respectively. By comparison, the pyrolytic gases MBM_PYGASSBAs_ record levels of C1 between [11.12 vol.% to 18.69 vol.%] vs. MBM_PYGAS_ ~16.05 vol.%, while C2 and higher and C3 and higher register 14.51 vol.% and 14.63 vol.%, on average, respectively, and 23.14 vol.% and 21.20 vol.%. Through GC investigations, high levels of a green gas, H_2_, were found, respectively, at 19.49 vol.% for MBM_PYGASNiSBA16_, and finally the energy ratio as a consequence of the evaluation performed was, respectively, 69.09 MJ/m^3^ (MBM_PYGAS_) vs. 43.23 MBM_PYGAS(SBAs)_ (Figure 10). The literature presents the MBM_PYGAS_ in a wide temperature range, from 400 °C to 650 °C, with energy values recorded below those of the gases developed in this study, respectively, ~50 MJ/m^3^ [19,52,53]. With the C-rich organic matter being subjected to the pyrolysis process, it was expected that the pyrolysis processes would end with a high CO_2_ content. By comparison with the gases developed through the pyrolysis processes of plastic waste—PPG [45], the energy value of MBM_PYGAS_, 99.83 MJ/m^3^ vs. 69.09 MJ/m^3^ (Figure 10), it is obviously much lower. This fact is explained by the initial composition of the raw materials, namely, if in the case of MBM, we have a composition rich in C, N, S, O, and H, in terms of plastic waste, their structure is based on a proportion of 98% of C and H, two energetic elements [38]. As a common element, for both PPG and MBM_PYGAS_, the majority gas was not CH_4_, which led to a high energy value of the pyrolysis gas, since CH_4_ is the gas with the lowest energy value in the hydrocarbon chain. The other special matrix subjected to the pyrolysis process, the sewage sludge (SS) [59], has a content of the pyrolysis gas, mostly in CH_4_, of ~33 vol.% vs. 15.47 vol.% in MBM_PYGAS_s. Despite the content of microplastics, traces of liquid fossil fuels and waste oils, the HHV of SS_PYGAS_ was lower than that of MBM_PYGAS_, which was 33.81 MJ/m^3^ compared to 69.09 MJ/m^3^, respectively. In the case of GeoGas—geothermal gas, NG—natural gas, SS Biogas—sewage sludge from wastewater treatment plants and MSW Biogas—municipal solid waste from a non-hazardous waste treatment station, the high content of CH_4_, as a major component, is reflected in a low HHV compared to that of MBM_PYGAS_ (Figure 10) [59]. The MBM_PYGAS_ type has a high CO_2_ content, a similar element to biogas, and SS and/or MSW from, respectively, ~16 vol.% to 50 vol.% vs. 35 vol.%. This phenomenon can be worrying from the perspective of greenhouse gases (GHGs) and the climate changes generated by them, but this impact can be considerably reduced to negligible by using adsorbent materials that have a high selective CO_2_ adsorption capacity and no affinity for hydrocarbons, greatly improving the final HHV of the “filtered” gas [60]. The level of H_2_S in the case of pyrolytic gases based on MBM is similar to other types of combustible gases, such as NG, biogas SS, and biogas MSW: <200 ppm.

There was an obvious affinity for the development of higher H_2_ concentrations when pyrolysis processes used SBAs: 6.68 vol.% vs. 3.28 vol.% (MBM_PYGAS_/UPs), on average. In particular, the catalytic pyrolytic processes performed, both under the influence of SBAs, led to higher CO + CO_2_ values compared to non-catalytic processes (UP): 38.50 vol% vs. 20.47 vol%, on average. The latter statement is directly reflected in the energy values, being inversely proportional to the values of CO + CO_2_ concentrations, respectively: 69.09 MJ/m^3^(MBM_PYGAS_) vs. 46.23 MJ/m^3^ (MBM_PYGAS(SBAs)_) (Table 4).

Table 4 presents also other physical gas parameters (lower calorific value, density and Wobbe number), specific to the alternative fuel analysis and corresponding to the pyrolytic gases developed in this study.

The MBM_PYGAS_ can be used directly, respectively, in an engine with thermal and/or electrical conversion. Another option, mixing in different concentrations (as in the case of biogas SS or hydrogen) with natural gas and introduced into the national network, is an example of good disposal practice in waste recovery.

### 3.6. MBM_PYCHAR_ and Selective Adsorbent Materials

The third reaction product of MBM pyrolysis processes was MBM_PYCHAR_. The present study sought an alternative solution to eliminate by using MBM_PYCHAR_ by creating selective adsorbent materials. The development of selective adsorbent materials from MBM_PYCHAR_ followed a previously used methodology [59]. The elementary investigations, CHNS, highlighted a number of interesting aspects regarding the compositional evolution of the residue obtained by MBM pyrolysis. Specifically, a decrease in C level for MBM_PYCHAR_ and oxidized MBM_PYCHAR_ can be observed, from about 20 wt.% to 6 wt.%, respectively. In the stage of the treatment of the solid residue with KOH and HCl, an increase in the C level can be observed up to ~21 wt.%, and after calcination to ~25 wt.%. This increase in the C level is offset by the loss of metals from KOH and HCl treatment (Table 5).

Generally, the nutrient content (Ca, Mg and P) increased with pyrolysis temperature, especially Ca. Calcium and Mg, having higher boiling point, were enriched in the final products. The concentration of heavy metals except Zn was below 17 mg/kg. Zinc, which is known to have moderate volatility, was partially released in the volatile fraction, decreasing from 536.65 mg/kg to 5 mg/kg at higher temperature, implying the possible association with sulphates or organic matter [59]. The chemical composition of MBM is influenced by the type of raw material, (i) bone and meat ratio, (ii) type of animal or bird, (iii) processing conditions. Regarding the chemical composition of MBM, it was observed that more than 90% of the composition was represented by Ca and P, with the Ca:P ratio being 2.28:1 (Table 2). The solid powder obtained from the processes of pyrolysis, oxidation, and treatment with KOH and HCL was subjected to the calcination process using a special cartridge.

In order to ensure the continuous flow of inert gas through the cartridge and to avoid blocking the formation of caverns/pores of future selective adsorbent materials, silver spheres were introduced.

**Figure 11:**
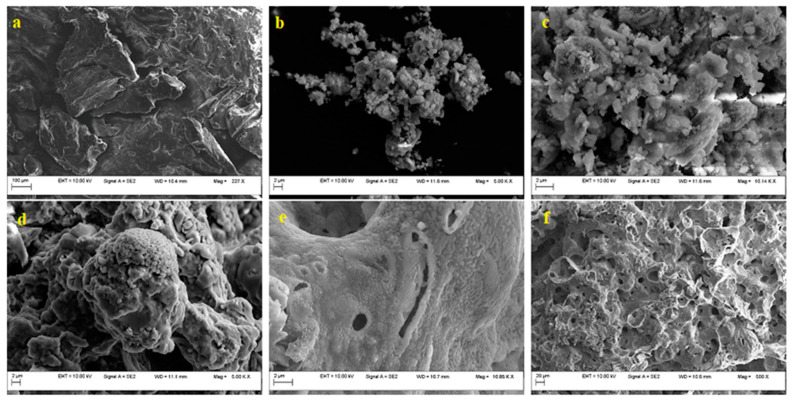
SEM images. Structural evolution depending on the treatment applied (**a**) MBM; (**b**) MBM_PYCHAR_ (UPs); (**c**) MBM_PYCHAR_ (oxidized); (**d**) MBM_PYCHAR_ (oxidized; KOH; HCl); (**e**) MBM_PYCHAR_ (oxidized; KOH; HCl; calcinated); (**f**) MBM_PYCHAR_ (oxidized; KOH; HCl; calcinated).

Following the chemical and thermal activation operations of MBM_PYCHAR_, it can be seen how the developed material, C ~25 wt.%, a coaly one, becomes a cavernous one, which recommends it for the area of selective adsorbent materials. The SEM micrographs (Figure 11) show the microstructural evolution, with very distinct morphology depending on the treatment applied: from a bulk (dense) appearance (Figure 11a), fine-grained (Figure 11b–e), to a very porous (not dense) microstructure (Figure 11f).

The adsorption/desorption capacity of the MBM_PYCHAR_-based selective adsorbent is strengthened by the results obtained from the BET-type investigation [61], which results in a high S_BET_ ~126 m^2^/g, superior to the data in the literature, respectively, at ~24 m^2^/g, ~48 m^2^/g, and ~40 m^2^/g [62] (Table 6).

**Figure 12:**
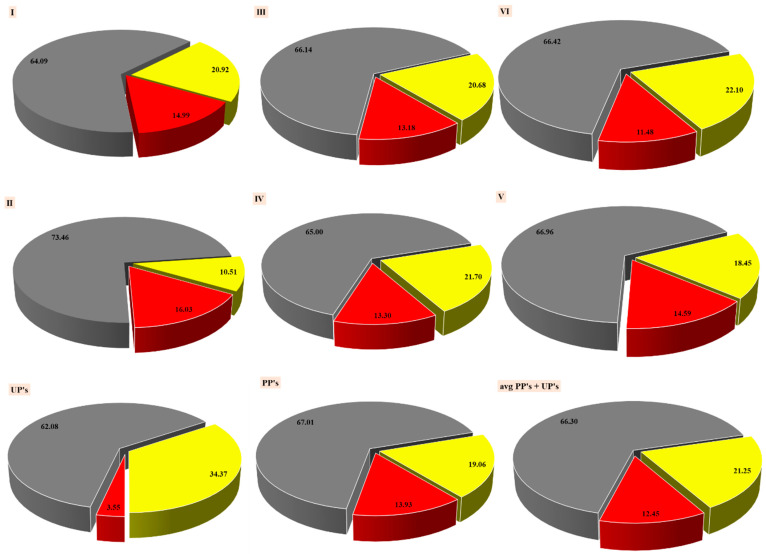
Comparative analysis (wt.%). Distribution of reaction products in the processes of catalytic and non-catalytic pyrolysis. Note: UPs—MBM non-catalytic processes; PPs—MBM pyrolysis processes on average (catalytic processes); MBM_PYGAS SBA3_—I; MBM_PYGAS SBA16_—II; MBM_PYGAS Fe-SBA3_—III; MBM_PYGAS Fe-SBA16_—IV; MBM_PYGAS Ni-SBA16_—V; MBM_PYGAS Ni-SBA3_—VI. Gray—MBM_PYCHAR_; red—MBM_PYOIL_; yellow—MBM_PYGAS_.

The analysis of the pyrolytic processes summarizes: (i) the highest affinity in developing MBM_PYOIL_, SBA-16 showed ~16.03 wt.%, and the lowest non-catalytic processes, UPs ~3.55 wt.% (Figure 12); (ii) Ni-SBA3 showed the highest affinity for developing MBM_PYGAS_ between catalytic processes: 22.10 wt.%. However, UP’s processes developed an even higher amount, ~34.37 wt.% (Figure 12); (iii) MBM_PYCHAR_’s concentration levels were >60 wt.%, as an average, and the highest was recorded by a catalytic process with SBA-16, 73.46 wt.% vs. 62.08 wt.%, UPs (Figure 12); (iv) catalytic processes compared to non-catalytic ones. They generated a higher percentage of MBM_PYCHAR_, 67.01 wt.% (SBAs) vs. 62.08 wt.% (UPs) (Figure 12); (v) catalytic processes compared to non-catalytic ones. They generated a higher percentage of MBM_PYOIL_, 13.93 wt.% (SBAs) vs. 3.55 wt.% (UPs) *(*Figure 12); (vi) catalytic processes compared to non-catalytic ones. They generated a lower percentage of MBM_PYGAS_, 34.37 wt.% (UPs)—19.06 wt.% (SBAs) (Figure 12); (vii) the most balanced pyrolysis process in terms of the oil:gas:char ratio, which was a catalytic type the with use of SBA-3, 14.99 wt.%:20.92 wt.%:64.09 wt.%; (viii) the high CO_2_ content, from 16 vol.% to 51 vol.%, existing in MBM_PYGAS_ may be an environmental problem, but through feasible solutions to retain it on carbonaceous supports, it can be subsequently used for the energy balancing of pyrolysis processes for various solid wastes; (ix) the use of catalysts had the role of generating, at the end of a pyrolysis process, one type of reaction product or another, depending on the economic/environmental interest, etc.

## 4. Conclusions

In this study, silica-based catalysts were prepared in order to be applied in MBM pyrolysis.

The obtained materials had high specific surfaces (from 268 up to 911 m^2^/g), mesoporous structure (with a medium pore diameter between 2.2 and 3.7 nm) and, also, uniform pore distribution, which constitute the vectors for their catalytic activities in the pyrolysis process.

For each of the developed pyrolysis processes (non-catalytic or catalytic), three reaction products (MBM_PYOIL_, MBM_PYGAS_, and MBM_PYCHAR)_ resulted in various proportions, caused by different drivers and individual variables.

The catalytic pyrolysis of MBM over SBA-3 and SBA-16 derivates constitutes a promising route for obtaining clean fuels and intermediates for the chemical and petrochemical industries. It is obvious that this method can be harnessed to re-use MBM waste which is prohibited to be used as animal feed according to European directives. This way, environmental problems arising from waste disposal can be reduced drastically while yielding useful products in the fuel oil range.

There was an obvious affinity for the development of higher H_2_ concentrations when pyrolysis processes used silica materials as catalysts. The catalytic pyrolytic processes led to higher values compared to non-catalytic processes for CO [7.20 vol.% vs. 3.71 vol.% on average] and CO_2_ [16.76 vol.% vs. 31.30 vol.% on average]. This was reflected in the higher heating values, which were inversely proportional to the values of CO and CO_2_ concentrations.

Additionally, the catalyst used in this study had minimal effect on the pyrolysis products. The aim of upgrading the quality of production through a catalyst reaction is less desirable for MBM feedstock. The oil and gas products did not demonstrate significant improvement in HHV and the biochar scenario did not use the catalytic scenario for upgrade.

Techno-economic analysis will be necessary in order to prognose, in advance, the feasibility of a catalytic pyrolysis plant. Additionally, this analysis can also consider the coupling of existing pyrolysis set-up to an additional upgrading step. Further assumptions of various parameters will be required, but the more assumptions are taken into consideration, the more the conclusions related to the techno-economic feasibility of the proposed set-up could divagate from the reality.

## Figures and Tables

**Table 1:** Elemental and EDX analysis for SBA-16 group.

Sample	C (wt.%)	H (wt.%)	O (wt.%)	Metal (wt.%)	Si (wt.%)
SBA-16	0.29	0.41	46.26	-	53.04
Ni-SBA-16	0.76	1.42	54.39	1.54 (EDX)/1.57 (SAA)	41.86
Fe-SBA-16	0.13	1.21	58.09	1.03 (EDX)/0.95 (SAA)	39.62

**Table 2:** Elemental and EDX analysis for SBA-3 group.

Sample	C (wt.%)	H (wt.%)	O (wt.%)	Metal (wt.%)	Si (wt.%)
SBA-3	0.40	0.64	50.25	-	48.71
Ni-SBA-3	1.65	1.42	54.16	2.50 (EDX)/2.55 (SAA)	40.22
Fe-SBA-3	0.55	1.01	51.59	0.98 (EDX)/1.00 (SAA)	45.75

**Table 3:** Content of heavy metals in MBM_PYOIL_, in μg/L.

Metals	Fuel Type							
	MBM_PYOIL_ *	PPO_PP_ [57]	PPO_HDPE_ [57]	PPO_LDPE_ [57]	PPO_PS_ [57]	Diesel [54]	Kerosene [54]	Gasoline [55]
Cd	0.01	0.12	0.34	0.05	0.07	15.00	13.30	16.80
Pb	1.55	0.09	0.53	0.06	0.07	10.10	4.10	2.40
Cr	0.81	1.97	2.49	3.11	10.03	8.60	3.30	5.40
Mn	0.07	0.29	8.11	0.01	0.37	<0.01	<0.01	<0.01
Co	0.02	0.21	1.39	0.07	0.16	<0.01	<0.01	<0.01
Ni	1.29	0.56	3.83	4.56	0.02	<0.01	<0.01	<0.01
Cu	<0.01	0.06	2.31	1.75	0.27	17.70	19.80	17.40
As	0.28	0.07	0.17	0.35	0.30	<0.01	<0.01	<0.01
Se	<0.01	1.61	1.72	2.40	2.12	<0.01	<0.01	<0.01
Hg	<0.01	<0.01	<0.01	<0.01	0.01	<0.01	<0.01	0.77
Rb	<0.01	0.17	0.01	0.21	0.22	<0.01	<0.01	<0.01
Sr	<0.01	0.06	0.72	0.05	0.15	<0.01	<0.01	<0.01

* non-catalytic process—developed in the present study.

**Table 4:** MBM_PYGAS_—alternative fuel characteristics.

	Parameters	UP	I	II	III	IV	V	VI
MBM_PYGAS_								
HHV (MJ/m^3^)		69.09	47.06	41.89	52.38	47.34	44.51	44.22
LHV (MJ/m^3^)		63.38	43.15	38.42	48.02	43.40	40.75	40.49
Density (kg/m^3^)		1.79	1.76	1.80	1.75	1.65	1.67	1.66
Wobbe number (MJ/m^3^)		58.72	40.34	35.46	45.06	41.96	39.15	39.03

**Table 5:** MBM_PYCHAR_—physicochemical characteristics of the pyrolysis residue.

	MBM_PYCHAR_				
Investigation	MBM	MBM_PYCHAR_ (UP’s)	MBM_PYCHAR_ (Oxidized)	MBM_PYCHAR_ (Oxidized; KOH; HCl)	MBM_PYCHAR_ (Oxidized; KOH; HCl; Calcinated)
*Elemental analysis by GC/TCD (wt.%)*					
C	20.31	10.35	6.85	20.85	24.88
N	4.14	1.74	1.32	3.46	3.51
H	3.28	0.79	0.67	3.49	1.87
S	0.52	0.76	0.81	<0.005	<0.005
*Gravimetric analysis (wt.%)*					
A	56.14	-	-	-	-
V	39.52	-	-	-	-
*Energy assessment by calorimetry (MJ/kg)*					
HHV	14.93	7.24	4.81	-	-
*Metal content by atomic absorption/AAS (mg/kg^−1^)*					
Pb	<17.00	<17.00	<17.00	<17.00	<17.00
Cu	15.33	31.32	16.52	70.66	96.32
P	161,801	172,830	37,612	1462	7837
Fe	74.08	163.99	101.01	398.25	479.94
Ni	<12.00	<12.00	<12.00	<12.00	<12.00
Mn	6.89	8.04	8.00	7.31	7.84
Zn	431.01	543.73	536.65	407.31	5.00
Ca	369,595	484,405	582,907	590	3186
Mg	3114	4900	4104	186	111
Na	8675	12,757	11,956	6813	4208

**Table 6:** BET characterization of MBM-based materials (S_BET_—Specific surface area; V—volume pore; D_v_—diameter of a pore).

Investigation	Material				
	MBM	MBM_PYCHAR_ (UP’s)	MBM_PYCHAR_ (Oxidized)	MBM_PYCHAR_ (Oxidized; KOH; HCl)	MBM_PYCHAR_-Based Selective Adsorbent
S_BET_ (m^2^/g)	1.021	117.113	0.748	2.441	125.647
V (cm^3^/g)	0.002	0.247	0.005	0.021	0.281
D_v_(r) (Å)	15.645	18.560	18.512	15.647	18.542

## Data Availability

Not applicable.
